# Quadruple Plasmon-Induced Transparency and Dynamic Tuning Based on Bilayer Graphene Terahertz Metamaterial

**DOI:** 10.3390/nano13172474

**Published:** 2023-09-01

**Authors:** Jiayu Zhang, Junyi Li, Shuxian Chen, Kunhua Wen, Wenjie Liu

**Affiliations:** 1 School of Physics and Optoelectronic Engineering, Guangdong University of Technology, Guangzhou 510006, China; 3220007487@mail2.gdut.edu.cn (J.Z.); 2112015089@mail2.gdut.edu.cn (J.L.); chensx@zen-semi.com (S.C.); 2 Institute of Advanced Photonics Technology, School of Information Engineering, Guangdong University of Technology, Guangzhou 510006, China; wjliu@gdut.edu.cn; 3 Key Laboratory of Photonic Technology for Integrated Sensing and Communication, Ministry of Education of China, Guangdong University of Technology, Guangzhou 510006, China; 4 Guangdong Provincial Key Laboratory of Information Photonics Technology, Guangdong University of Technology, Guangzhou 510006, China

**Keywords:** plasmon-induced transparency, optical switch, Fermi level, graphene

## Abstract

This study proposes a terahertz metamaterial structure composed of a silicon–graphene–silicon sandwich, aiming to achieve quadruple plasmon-induced transparency (PIT). This phenomenon arises from the interaction coupling of bright–dark modes within the structure. The results obtained from the coupled mode theory (CMT) calculations align with the simulations ones using the finite difference time domain (FDTD) method. Based on the electric field distributions at the resonant frequencies of the five bright modes, it is found that the energy localizations of the original five bright modes undergo diffusion and transfer under the influence of the dark mode. Additionally, the impact of the Fermi level of graphene on the transmission spectrum is discussed. The results reveal that the modulation depths (MDs) of 94.0%, 92.48%, 93.54%, 96.54%, 97.51%, 92.86%, 94.82%, and 88.20%, with corresponding insertion losses (ILs) of 0.52 dB, 0.98 dB, 1.37 dB, 0.70 dB, 0.43 dB, 0.63 dB, 0.16 dB, and 0.17 dB at the specific frequencies, are obtained, achieving multiple switching effects. This model holds significant potential for applications in versatile modulators and optical switches in the terahertz range.

## 1. Introduction

Surface Plasmon Polaritons (SPPs) [1], which are collective resonances produced by the interaction of incident photons and surface electrons [2,3], have been extensively studied in metallic and graphene materials [4,5,6,7,8]. Graphene, a tunable two-dimensional hexagonal lattice material, exhibits similar dielectric constants to noble metals in the terahertz and infrared wavelengths [9]. Recently, researchers have noted the occurrence of the PIT phenomenon in both graphene [10,11,12] and metallic structures [13]. However, those waveguide structures utilizing noble metals only allow for static adjustments and suffer from significant losses [14]. In comparison, graphene offers significant advantages with dynamic modulation through gate voltage tuning, making it highly versatile [15,16]. Because of its low loss [17,18,19], excellent dynamic tunability, and significant local field enhancement effect [18,19,20,21], graphene finds crucial applications in optical sensing [22,23,24], optical switching [25,26], optical storage [27,28], etc. 

Graphene-excited surface plasmons show robust localized field enhancement effects in the terahertz and infrared wavelengths. The plasmon excitations on the surface of graphene can generate plasmon-induced transparency (PIT) [29], which is a phenomenon similar to the electromagnetically induced transparency (EIT) [30,31] caused by atomic coherence. However, due to the challenging experimental conditions and high costs associated with EIT, it is not conducive to practical applications [32]. PIT is a transparent phenomenon produced in classical systems by destructive interference. It shares similarities with EIT, but the required experimental conditions are not as stringent as those of EIT [33,34]. Recently, PIT has been gaining significant attention among numerous researchers, owing to its unique characteristics [35,36,37]. Studies have indicated that the interplay of bright and dark modes can generate the PIT effect [38]. Bright modes, which are directly excited by the incident light, induce resonance at specific frequencies, thereby causing a continuous transmission dip within the transmission spectra. Conversely, dark modes, being unable to be directly stimulated by incident light, typically do not impact the spectra. However, in those designed bright–dark mode symbiosis structures, the dark mode is brought into action through an optical field created by the interaction of the incident light and the bright mode, leading to anomalous light transmission and a PIT window.

In this study, we present a dual-layer graphene waveguide structure composed of five graphene ribbons (M1, M2, M3, M4, M5) and double vertical graphene ribbons (DVRG) to achieve multiple PIT windows. The proposed structure is a multilayer terahertz metamaterial, in which a silicon substrate is sandwiched between two graphene layers, each connected to its respective electrodes, respectively. Furthermore, the destructive interference between the structure’s four bright modes and one dark mode results in significant PIT. The theoretical results of quadruple PIT are fitted using the coupled mode theory (CMT), which shows high consistency with the outcomes of the finite difference time domain (FDTD) simulations. Importantly, excellent optical modulation characteristics are achieved for our structure, since the maximum and minimum modulation depths (MDs) reach 97.51% and 88.20%, corresponding to the insertion losses (ILs) of 0.43 dB and 0.17 dB, respectively. Therefore, our proposed structure offers significant prospects in areas such as optical switching and modulation.

## 2. Simulation Model

**Figure 1:**
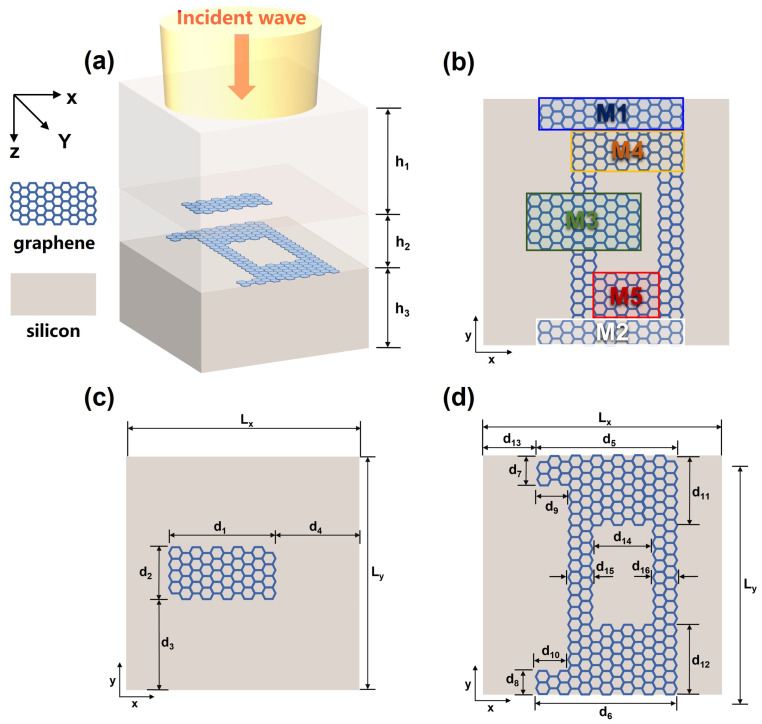
(**a**) A 3D diagram presents the entirety of the proposed graphene structure. (**b**) An overhead perspective providing a top view of the graphene arrangement. (**c**) Diagram of the graphene structure on the upper layer in the z-direction of the structure. (**d**) Diagram of the graphene structure on the lower layer in the z-direction of the structure.

The three-dimensional view depicted in Figure 1a showcases a terahertz metamaterial structure consisting of a double-layer graphene. The substrate of the structure is made of silicon, serving as the supporting layer, with a thickness of 0.45μm. The upper layer consists of a single rectangular graphene (M1), while the lower layer is composed of four rectangular graphene sheets (M2, M3, M4, M5) and double vertical rectangular graphene (DVRG) sheets, with each graphene layer having a thickness of 1 nm. Figure 1b provides a distinct depiction of the top view of the proposed metamaterial structure. Figure 1c,d provide the detailed parameters of the upper and lower layers of graphene. The geometric parameters are as follows: $d_{1}=0.9\;\mu m$, $d_{2}=0.3\;\mu m$, $\;d_{3}=d_{4}=0.8\;\mu m$, $d_{5}=d_{6}=1.1\;\mu m$, $d_{7}=d_{8}=d_{9}=d_{10}=0.2\;\mu m$, $d_{11}=d_{12}=0.6\;\mu m$, $d_{13}=0.5\;\mu m$, $d_{14}=0.7\;\mu m$, $d_{15}=d_{16}=0.2\;\mu m$, $L_{x}=L_{y}=2\;\mu m$, $h_{1}=0.2\;\mu m$, $h_{2}=0.1\;\mu m$, $h_{3}=0.15\;\mu m$. The incident plane wave, serving as the excitation source, propagates in the negative direction of the z-axis, perpendicular to the x–y plane. Monitors are positioned at a distance of 0.15 μm above the bottom of the silicon substrate. In the X and Y directions, periodic boundary conditions (PBC) are used and in the Z direction, a perfect matching layer (PML) is applied. PBC is suitable for periodic structures, reducing computational resource requirements and avoiding edge effects. Meanwhile, PML effectively absorbs reflected waves, ensuring the accuracy of simulation results. The mesh step sizes in the x and y directions are set to 0.1 µm, while the mesh step size in the z direction is set to 0.02 µm, with a utilization of 22.5 mesh nodes vertically. The simulation time is set to 15,000 fs. The Fermi level of the graphene ribbon is 0.6 eV.

Within the context of our proposed metamaterial structure, Molecular Beam Epitaxy (MBE) presents a promising approach for fabricating the upper Si regions. MBE is capable of growing crystalline thin films with excellent layer thickness and material quality control. This technique involves the precise deposition of atoms or molecules onto a substrate surface under ultra-high vacuum conditions. By finely adjusting growth parameters such as temperature, deposition rate, and silicon precursor flux, the controlled growth of Si layers with nanoscale thickness can be achieved.

The Kubo model stands as a well-established theoretical framework in the realm of condensed matter physics. It is harnessed to ascertain the electrical conductivity of graphene, which can be formulated as follows [38]:

(1) $$\sigma \left(\omega \right)=\sigma_{intra}+\sigma_{inter}$$

(2) $$\sigma_{intra}=\frac{2ie^{2}k_{B}T}{\pi \hbar^{2}\left(\omega +ir^{-1}\right)}\ln\left[2\cosh(\frac{E_{F}}{2k_{B}T})\right]$$

(3) $$\sigma_{inter}=\frac{e^{2}}{4\hbar^{2}}\left[\frac{1}{2}+\frac{1}{\pi }\arctan\left(\frac{\hbar \omega -2E_{F}}{2k_{B}T}\right)\right]-\frac{e^{2}}{4\hbar^{2}}\left[\frac{i}{2\pi }\ln\frac{{\left(\hbar \omega +2E_{F}\right)}^{2}}{{\left(\hbar \omega +2E_{F}\right)}^{2}+4{\left(k_{B}T\right)}^{2}}\right]$$

where $e,\;\hbar ,\;k_{B},\;\omega ,\;E_{F}$ stand for the electron charge, the reduced Planck constant, the Boltzmann constant, the angular frequency of incident light and the Fermi level of graphene, respectively. μ is carrier mobility, and $v_{F}=10^{6}\;ms^{-1}$ denotes the Fermi velocity. From $\tau =\mu E_{F}/ev_{F}^{2}$ [39], the carrier relaxation time τ can be deduced. The ambient temperature is configured as T=300 K. By adjusting the bias voltage, the Fermi level of graphene $E_{F}$ can be modulated from 0.6 eV to 0.72 eV. Under the conditions established in this study, $\sigma_{inter}$ can be disregarded, so σ can be obtained [40,41]:

(4) $$\sigma \left(\omega \right)=\frac{e^{2}E_{F}}{\pi \hbar^{2}}\frac{i}{\omega +i\tau^{-1}}$$

In the proposed graphene metamaterial structure, with silicon as the substrate, the propagation constant β and effective refractive index $n_{eff}$ are as follows [38,42]:

(5) $$\frac{\varepsilon_{si}}{\sqrt{\beta^{2}-\varepsilon_{si}k_{0}{}^{2}}}+\frac{\varepsilon_{air}}{\sqrt{\beta^{2}-\varepsilon_{air}k_{0}{}^{2}}}=-\frac{i\sigma }{\omega \varepsilon_{0}}$$

(6) $$n_{eff}=\frac{\beta }{k_{0}}$$

where $\varepsilon_{si},\;\varepsilon_{air},\;\mathrm{and}\;\varepsilon_{0}$ present the relative dielectric constants of silicon, air and vacuum dielectric, respectively. $k_{0}$ and $\eta_{0}$ correspond to the wave number in free space and the inherent impedance, respectively.

## 3. Results of Simulation and Theoretical Analysis

**Figure 2:**
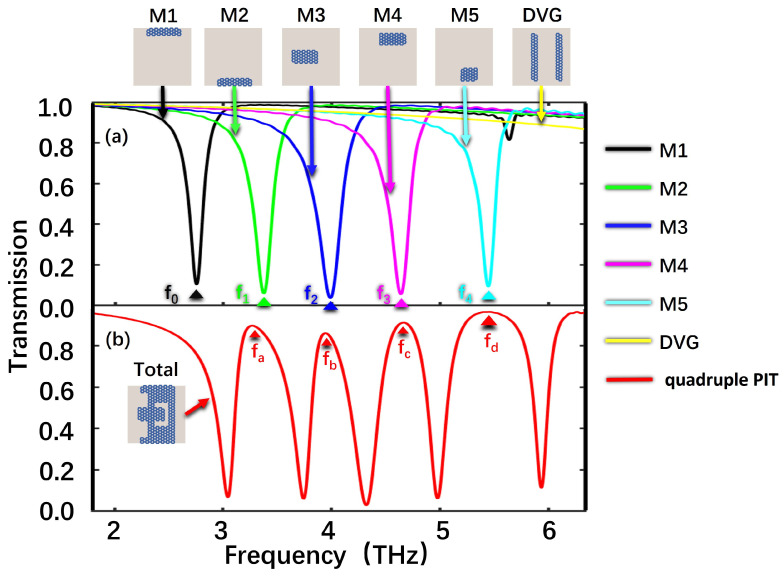
(**a**) The transmission spectra of individual M1 (black line), M2 (green line), M3 (dark blue line), M4 (purple line), and M5 (light blue line). (**b**) The transmission spectra of the entire structure when the structure is under direct incident light excitation. Here, the E_F_ = 0.6 eV.

The FDTD simulation process involves numerically solving Maxwell’s equations using a grid-based approach. It discretizes both time and space, allowing us to track the evolution of electromagnetic fields over discrete time steps, enabling analysis of its optical behavior and properties. In the simulation, the graphene layer is discretized into grid cells both in time and space. The electromagnetic field interacts with the graphene layer based on its material properties, and the FDTD algorithm iteratively solves Maxwell’s equations in a timely manner to calculate the propagation of the electromagnetic field and its interaction with the graphene layer. To elucidate the physical mechanism behind the emergence of the quadruple PIT phenomenon, the proposed structure is simulated using the FDTD method, and the transmission spectra are depicted in Figure 2. When an x-polarized beam is vertically incident on the structure, different graphene structures exhibit distinct Lorentz curves. In Figure 2a, the M1, M2, M3, M4, and M5 structures generate five Lorentz modes, considered as the bright modes, which can be readily stimulated by the incident plane wave. The resonant frequencies of these bright modes, denoted as $f_{0}$, $f_{1}$, $f_{2}$, $f_{3}$, and $f_{4}$, are 2.75 THz, 3.37 THz, 3.98 THz, 4.63 THz, and 5.44 THz, respectively. When the incident light is launched on the DVRG, similar bright modes are hardly excited, but the DVRG generates a mode with a transmission of approximately 1, which serves as the dark mode. The dark mode is incapable of direct excitation by the incident plane wave, yet it engages with the localized field generated by the bright modes. Therefore, the destructive interferences between the five bright modes and the dark mode lead to a pronounced quadruple PIT phenomenon, as shown by the solid red curve, indicating the formation of the quadruple PIT windows. The four PIT peaks are labeled as $f_{a}$, $f_{b}$, $f_{c}$, and $f_{d}$ from left to right in Figure 2b with the red curve. At the resonant frequencies of the bright modes ($f_{0}$, $f_{1}$, $f_{2}$, $f_{3}$, and $f_{4}$), the originally bright modes transform into the states with transmission rates close to 1, as confirmed in Figure 2a,b.

**Figure 3:**
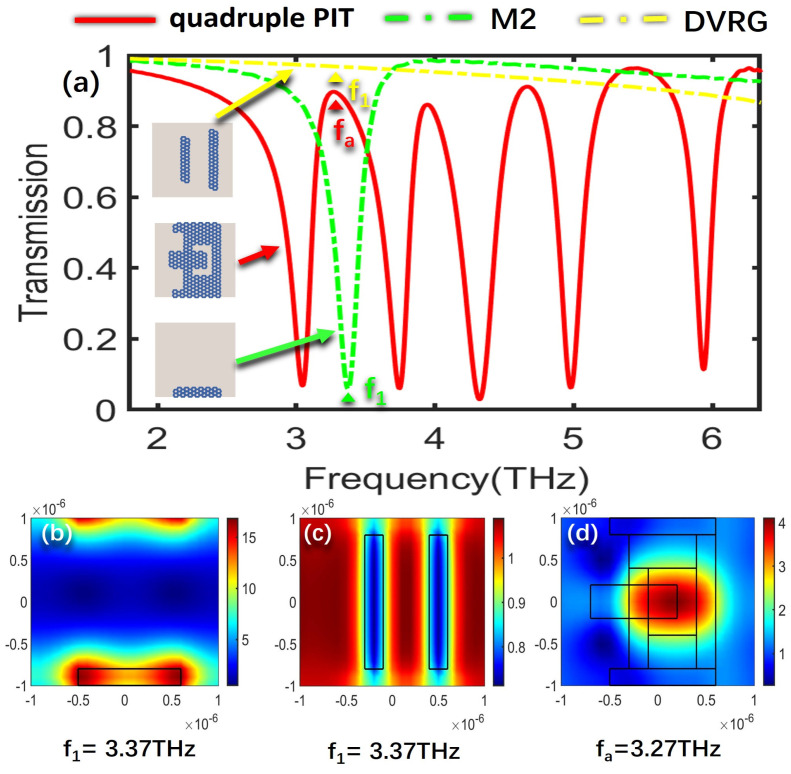
(**a**) Transmission spectra for the scenarios involving only M2, DVRG, and the entire structure. (**b**,**c**) The spatial distribution of the electric field within structures M2 and DVRG at the resonant frequency f_1_. (**d**) Electric field distribution of the entire structure at f_a_.

**Figure 4:**
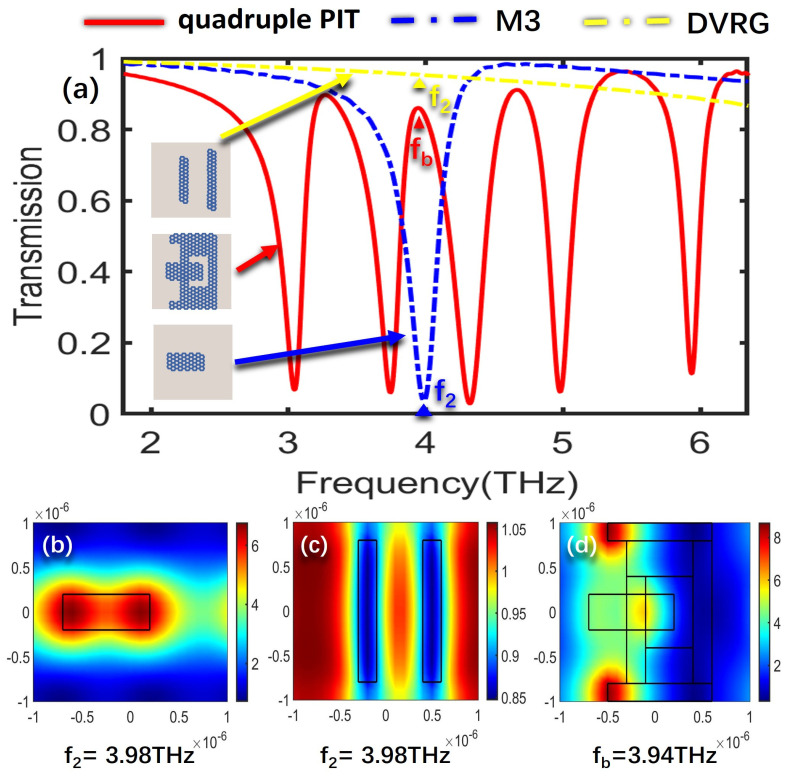
(**a**) Transmission spectra for the scenarios involving only M3, DVRG, and the entire structure. (**b**,**c**) The spatial distribution of the electric field within structures M3 and DVRG at the resonant frequency f_2_. (**d**) Electric field distribution of the entire structure at f_b_.

**Figure 5:**
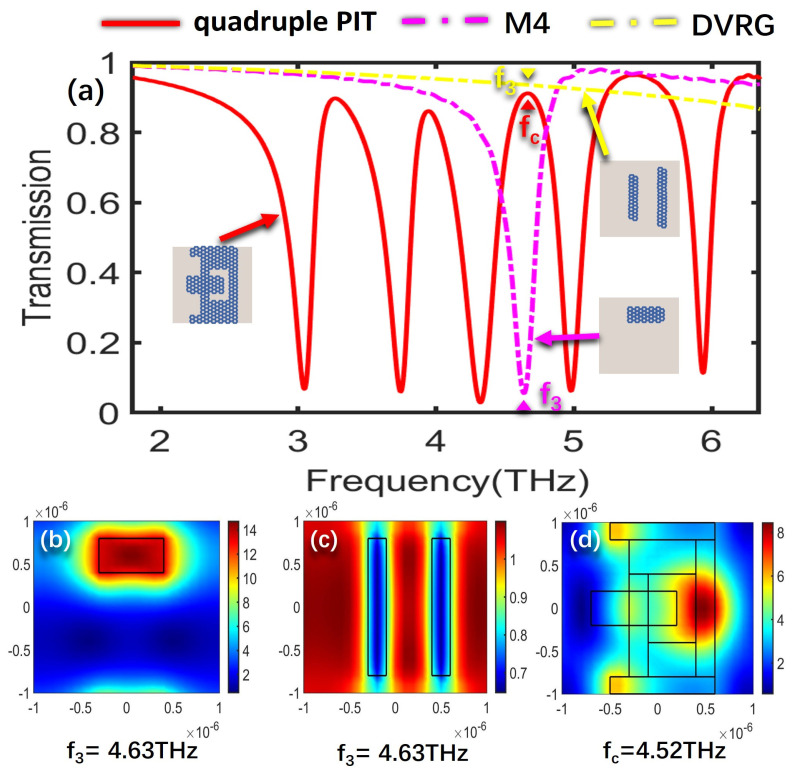
(**a**) Transmission spectra for the scenarios involving only M4, DVRG, and the entire structure. (**b**,**c**) The spatial distribution of the electric field within structures M4 and DVRG at the resonant frequency f_3_. (**d**) Electric field distribution of the entire structure at f_c_.

**Figure 6:**
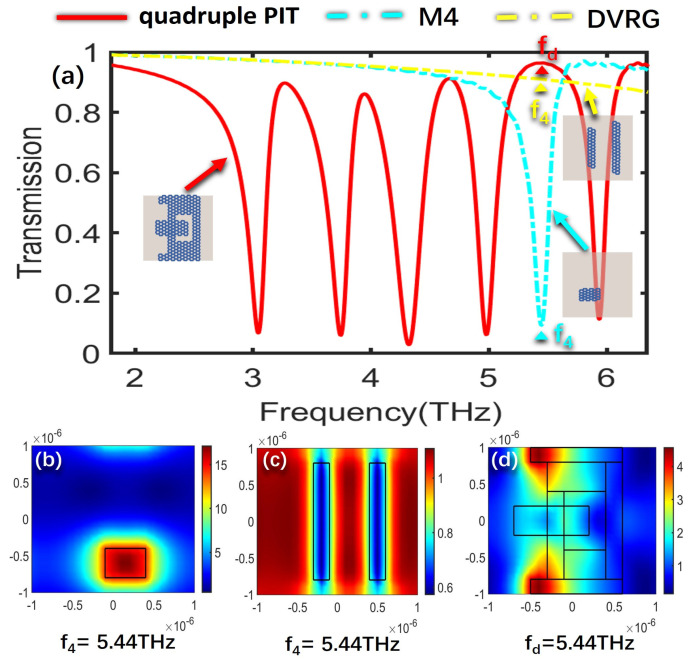
(**a**) Transmission spectra for the scenarios involving only M5, DVRG, and the entire structure. (**b**,**c**) The spatial distribution of the electric field within structures M5 and DVRG at the resonant frequency f_4_. (**d**) Electric field distribution of the entire structure at f_d_.

**Figure 7:**
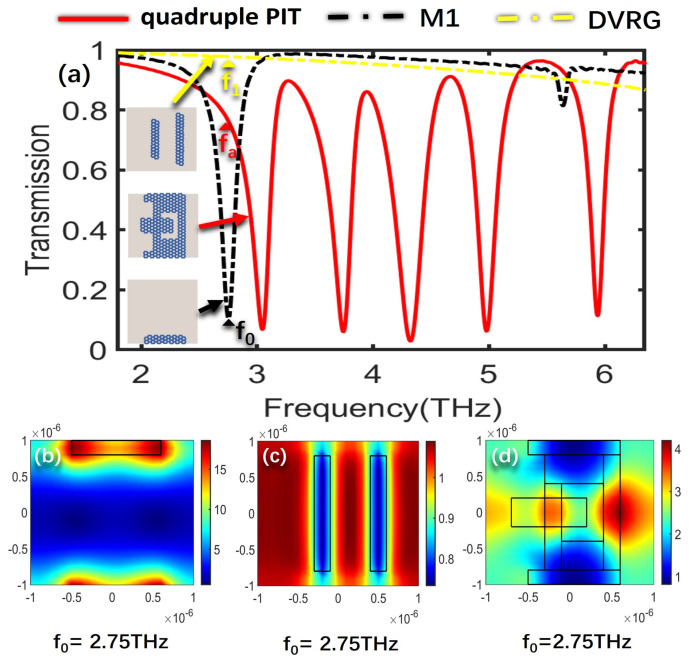
(**a**) Transmission spectra for the scenarios involving only M1, DVRG, and the entire structure. (**b**–**d**) The spatial distribution of the electric field within structures M4, DVRG, and the entire structure at the resonant frequency f_0_.

**Figure 8:**
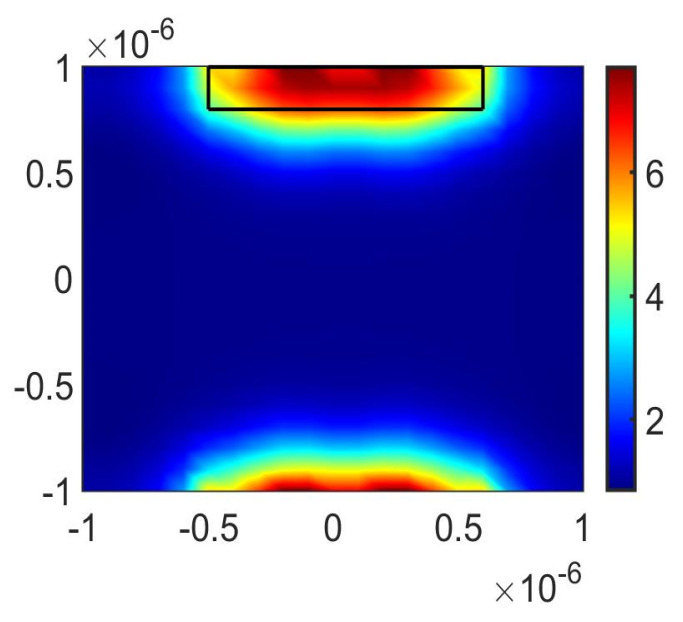
The spatial distribution of the electric field within structure M1 at 5.64 THz.

To further delve into the underlying formation mechanism that governs the creation of the quadruple PIT, the electric field distributions of the bright modes, the dark mode, and the overall structure at the resonant frequencies of the four PIT windows are analyzed, respectively. The SPPs’ excitation of graphene determines the intensity of the electric field, where a higher excitation level results in stronger electric field energy. Figure 3a illustrates the transmission spectra for the cases of only M2, DVRG, and the entire structure. Figure 3b,c represent the electric fields of the bright mode M2 and the dark mode DVRG at the resonant frequency of 3.37 THz, respectively. In Figure 3b, the electric field distribution at the edges of graphene appears in a darker shade of red, indicating higher energy levels. Graphene strongly couples with the incident plane wave for generating the bright mode, and then the energy of the incident wave tightly binds with the graphene. In Figure 3c, when only two vertical graphene strips DVRG are presented, the electric field energy primarily localizes outside the graphene strips, with a very weak energy level. Within the terahertz range, it exhibits no significant response to the incident plane wave, behaving as a dark mode. Figure 3d represents the spatial distribution of the electric field within the entire structure at the first resonant peak $f_{a}$. The energy of the bright mode M2 experiences significant attenuation and transfers to the dark mode DVRG, induced by the near-field coupling between the bright and dark modes. At the resonance frequency of 3.27 THz, a distinctive and clearly pronounced transparency window emerges as a result of the destructive interference occurring between the two distinct modes. Figure 4a depicts the transmission spectra for the scenarios involving only M3, DVRG, and the entire structure. Figure 4b,c depict the spatial distribution of the electric field within the bright mode M3 and the dark mode DVRG at the resonant frequency of 3.98 THz. It can be observed that when M3 and DVRG exist independently, energy localization occurs independently under the influence of the incident light. In Figure 4b, the energy is mainly contributed by the rectangular graphene strip. In Figure 4c, the weaker energy is localized along the edges of the two vertical graphene strips. Referring to Figure 4d, which depicts the energy distribution of the electric field across the entire structure at the second resonance peak, the enhanced electric field is mostly distributed on the M1, M2 and DVRG, while the energy within the M3 region diminishes. This demonstrates the transfer of electric field energy from the M3 structure to the DVRG and M2, induced by the resonant interaction between the bright and dark modes. Figure 5 depicts the emergence of the second notable PIT transparency window, resulting from the destructive interference between the two modes, which is similar to the previous analysis. Figure 5a illustrates the transmission spectra for the cases of only M4, DVRG, and the entire structure. Figure 5b,c represent the electric fields of the bright mode M4 and the dark mode DVRG at the resonant frequency of 4.63 THz. At the third PIT transparency window, the dark mode is indirectly excited, and the electric field energy primarily propagates from the M4 structure to the DVRG, as depicted in Figure 5d. Figure 6a illustrates the transmission spectra for the cases of only M5, DVRG, and the entire structure. Figure 6b illustrates the direct excitation of M5 by the incident light, resulting in high electric field energy. At the fourth resonance peak $f_{d}$, the electric field on the M5 structure weakens, and the energy transfers to the M1 and M2 structures and weakly couples with the vertical graphene strips. Furthermore, the bright mode M1 at the resonant frequency of 2.75 THz undergoes destructive interference with other graphene structures, causing the transmittance changed from ~0 to ~1. Figure 7a illustrates the transmission spectra for the cases of only M1, DVRG, and the entire structure. Figure 7b indicates that the energy at 2.75 THz is primarily contributed by M1. Figure 7c demonstrates that the edges of the vertical graphene strips DVRG exhibit a conspicuous presence of extremely weak electric field energy, and a low energy level across the entire structure is revealed in Figure 7d. The energy on M1 is significantly attenuated, and the energy undergoes transfer and diffusion, primarily weakly localizing outside the vertical graphene strips DVRG and graphene structures. Therefore, under the influence of the incident plane waves, the bright mode M1 at the resonant frequency of 2.75 THz experiences destructive interference with the remaining graphene structures. This is investigated in the transmission spectrum with quadruple PIT, where the absorption peak disappears and the transmittance increases from 0.10 to 0.76. In Figure 8, the electric field energy is concentrated at the central local position of M1, and the energy level is relatively low. This phenomenon indicates that at the frequency of approximately 5.64 THz, the geometrical features of structure M1 resonate with the wavelength of the incident electromagnetic wave, leading to the concentration of electric field energy in specific regions. Due to the lower energy level, this resonance behavior is not as pronounced as the main peak. Therefore, a smaller peak appears in the transmission spectrum line represented by the black line in Figure 2a.

Based on the analysis process above, it can be understood that in the absence of the dark mode, the energy of the five bright modes remains independently localized. However, with the inclusion of the dark mode, the destructive interferences between the dark mode and the five bright modes give rise to the formation of quadruple PIT. The originally localized bright modes undergo diffusion and transfer under the influence of the dark mode, resulting in the enhanced energy of the dark mode. This mechanism plays a crucial role in the emergence of the quadruple PIT effect.

## 4. Math Method

**Figure 9:**
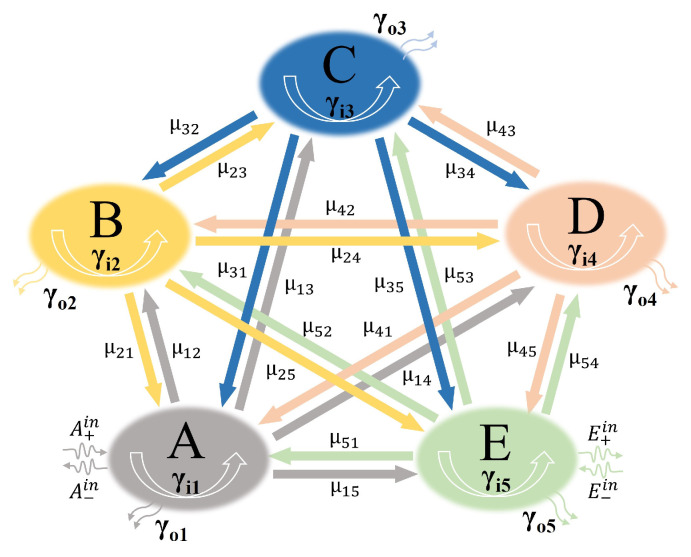
Theoretical schematic depicting the coupling among the resonant modes in the proposed graphene metamaterial structure.

To validate the FDTD simulation results, the CMT is employed to fit the FDTD simulation results. The theoretical coupling diagram derived from the CMT is depicted in Figure 9, where elements A, B, C, D, and E represent the five resonators (corresponding to M2, M3, M4, M5, and DVRG, respectively), and the amplitudes of the resonator modes are labeled as “a, b, c, d, and e”, respectively. The superscripts “in” and “out” of “$A_{\pm }^{\mathrm{in}/\mathrm{out}},\;B_{\pm }^{\mathrm{in}/\mathrm{out}},\;C_{\pm }^{\mathrm{in}/\mathrm{out}},\;D_{\pm }^{\mathrm{in}/\mathrm{out}},\;\mathrm{and}\;E_{\pm }^{\mathrm{in}/\mathrm{out}}$” are respectively the input and output waves of the resonator. The subscripts “+” and “−” of “$A_{\pm }^{\mathrm{in}/\mathrm{out}},\;B_{\pm }^{\mathrm{in}/\mathrm{out}},\;C_{\pm }^{\mathrm{in}/\mathrm{out}},\;D_{\pm }^{\mathrm{in}/\mathrm{out}},\;\mathrm{and}\;E_{\pm }^{\mathrm{in}/\mathrm{out}}$” represent the positive and negative directions of wave propagation, respectively. Drawing upon the concepts of CMT [43,44], the mathematical depiction that elucidates the interconnections among the various resonant modes finds its expression as follows:

(7) $$\left(\begin{array}{ccccc} \gamma_{1} & -i\mu_{12} & -i\mu_{13} & -i\mu_{14} & -i\mu_{15}\\ -i\mu_{21} & \gamma_{2} & -i\mu_{23} & -i\mu_{24} & -i\mu_{25}\\ -i\mu_{31} & -i\mu_{32} & \gamma_{3} & -i\mu_{34} & -i\mu_{45}\\ -i\mu_{41} & -i\mu_{42} & -i\mu_{43} & \gamma_{4} & -i\mu_{45}\\ -i\mu_{51} & -i\mu_{52} & -i\mu_{53} & -i\mu_{54} & \gamma_{5} \end{array}\right).\left(\begin{array}{c} a\\ b\\ c\\ d\\ e \end{array}\right)=\left(\begin{array}{ccccc} -\gamma_{o1}^{1/2} & 0 & 0 & 0 & 0\\ 0 & -\gamma_{o2}^{1/2} & 0 & 0 & 0\\ 0 & 0 & -\gamma_{o3}^{1/2} & 0 & 0\\ 0 & 0 & 0 & -\gamma_{o4}^{1/2} & 0\\ 0 & 0 & 0 & 0 & -\gamma_{o5}^{1/2} \end{array}\right).\left(\begin{array}{c} A_{+}^{in}+A_{-}^{in}\\ B_{+}^{in}+B_{-}^{in}\\ C_{+}^{in}+C_{-}^{in}\\ D_{+}^{in}+D_{-}^{in}\\ E_{+}^{in}+E_{-}^{in} \end{array}\right)$$

where $\mu_{mn}$ denotes the mutual coupling coefficients that interconnect the five resonators (m, n=1, 2, 3, 4, 5, m≠n). The variable $\gamma_{n}$ (with n values of 1, 2, 3, 4, 5) is contingent upon the resonant angular frequency $\omega_{n}$ and is explicitly characterized by the equation $\gamma_{n}=i\omega -i\omega_{n}-\gamma_{in}-\gamma_{on}$ (for n=1, 2, 3, 4, 5), where the inter-loss coefficient $\gamma_{in}$ is determined by $\gamma_{in}=\omega_{n}/(2Q_{in})$ and the external loss coefficient $\gamma_{on}$ is obtained by $\gamma_{on}=\omega_{n}/(2Q_{on})$. In addition, the overall quality factor $Q_{tn}$, the inherent loss quality factor $Q_{in}$, and the exterior loss quality factor $Q_{on}$ for the nth resonant mode adhere to the correlation $1/Q_{tn}=1/Q_{in}+1/Q_{on}\;\left(n=1,\;2,\;3,\;4,\;5\right)$, where $Q_{tn}=f/\Delta f$ represents the complete quality factor of the nth mode (f stands for resonance frequency and Δf signifies the full width at half maximum). The calculation of $Q_{in}$ involves the formulation $Q_{in}=Re\left(n_{eff}\right)\;/\;Im\left(n_{eff}\right)$ [40]. Abiding by the law of energy conservation, the correlation between the input wave and the output wave can be articulated as follows:

(8) $$B_{+}^{\mathrm{in}}=A_{+}^{\mathrm{out}}\cdot \exp(i\varphi_{1}),A_{-}^{\mathrm{in}}=B_{-}^{\mathrm{out}}\cdot \exp(i\varphi_{1}),$$

(9) $$C_{+}^{\mathrm{in}}=B_{+}^{\mathrm{out}}\cdot \exp(i\varphi_{2}),B_{-}^{\mathrm{in}}=C_{-}^{\mathrm{out}}\cdot \exp(i\varphi_{2}),$$

(10) $$D_{+}^{\mathrm{in}}=C_{+}^{\mathrm{out}}\cdot \exp(i\varphi_{3}),C_{-}^{\mathrm{in}}=D_{-}^{\mathrm{out}}\cdot \exp(i\varphi_{3}),$$

(11) $$E_{+}^{\mathrm{in}}=D_{+}^{\mathrm{out}}\cdot \exp(i\varphi_{4}),D_{-}^{\mathrm{in}}=E_{-}^{\mathrm{out}}\cdot \exp(i\varphi_{4}),$$

(12) $$A_{+}^{out}=A_{+}^{in}-\gamma_{o1}^{1/2}\cdot a,A_{-}^{out}=A_{-}^{in}-\gamma_{o1}^{1/2}\cdot a,$$

(13) $$B_{+}^{out}=B_{+}^{in}-\gamma_{o2}^{1/2}\cdot b,B_{-}^{out}=B_{-}^{in}-\gamma_{o2}^{1/2}\cdot b,$$

(14) $$C_{+}^{out}=C_{+}^{in}-\gamma_{o3}^{1/2}\cdot c,C_{-}^{out}=C_{-}^{in}-\gamma_{o3}^{1/2}\cdot c,$$

(15) $$D_{+}^{out}=D_{+}^{in}-\gamma_{o4}^{1/2}\cdot d,D_{-}^{out}=D_{-}^{in}-\gamma_{o4}^{1/2}\cdot d,$$

(16) $$E_{+}^{out}=E_{+}^{in}-\gamma_{o5}^{1/2}\cdot e,E_{-}^{out}=E_{-}^{in}-\gamma_{o5}^{1/2}\cdot e,$$

where $\phi_{1}$, $\phi_{2}$, $\phi_{3}$, and $\phi_{4}$ denote the phase differences between A and B, B and C, C and D, D and E, respectively. Meanwhile, the values of $\phi_{n}\;\left(n=1,\;2,\;3,\;4\right)$ can be derived utilizing the association $\phi_{n}=Re\left(\beta \right)d_{n}=\omega d_{n}Re\left(n_{eff}\right)/c$, where $d_{n}$ designates the distance between layers of graphene.

In addition, there is an absence of input wave propagating in the opposing direction in E, thus

(17) $$E_{-}^{in}=0.$$

According to Equations (7)–(17), the complete system’s transmission coefficient can be represented as follows:

(18) $$\begin{array}{c} t=\frac{E_{+}^{out}}{A_{+}^{in}}=1+\frac{\gamma_{o5}}{\gamma_{5}}+\left(\sqrt{\gamma_{o1}}+\sqrt{\gamma_{o5}}\frac{x_{51}}{r_{5}}\right)t_{1}+\left(\sqrt{\gamma_{o2}}+\sqrt{\gamma_{o5}}\frac{x_{52}}{r_{5}}\right)t_{2}\\ +\left(\sqrt{\gamma_{o3}}+\sqrt{\gamma_{o5}}\frac{x_{53}}{r_{5}}\right)t_{3}+\left(\sqrt{\gamma_{o4}}+\sqrt{\gamma_{o5}}\frac{x_{54}}{r_{5}}\right)t_{4} \end{array}$$

where

(19) $$t_{1}=-\frac{t_{a}}{t_{c}},$$

(20) $$t_{2}=-\frac{t_{b}}{t_{c}},$$

(21) $$t_{3}=\frac{lt+ps}{kp-lo}+\frac{ip-lm}{kp-lo}.\frac{t_{a}}{t_{c}}+\frac{jp-ln}{kp-lo}.\frac{t_{b}}{t_{c}},$$

(22) $$t_{4}=\frac{o(lt+ps)-t(lo-kp)}{p(kp-lo)}+\frac{m(lo-kp)+o(ip-lm)}{p(kp-lo)}.\frac{t_{a}}{t_{c}}+\frac{n(lo-kp)+o(jp-ln)}{p(kp-lo)}.\frac{t_{b}}{t_{c}},$$

(23) $$\begin{array}{ll} t_{a} & =\left[\left(hs+lr\right)\left(ch+dg\right)-\left(hq-dr\right)\left(hk-gl\right)\right]\times \left[\left(kp-lo\right)\left(fl+jh\right)-\left(jp-ln\right)\left(hk-gl\right)\right]\\ & +\left[\left(\mathrm{df}+bh\right)\left(hk-gl\right)+\left(ch+dg\right)\left(fl+jh\right)\right]\times \left[\left(kp-lo\right)\left(el+hi\right)-\left(ip-lm\right)\left(hk-gl\right)\right], \end{array}$$

(24) $$\begin{array}{l} a=\gamma_{1}x_{25}+x_{15}x_{21},b=\gamma_{2}x_{15}+x_{12}x_{25},c=x_{15}x_{23}+x_{13}x_{25},d=x_{15}x_{24}+x_{14}x_{25},\\ e=x_{25}x_{31}+x_{21}x_{35},f=\gamma_{2}x_{35}+x_{25}x_{32},g=\gamma_{3}x_{25}+x_{23}x_{35},h=x_{25}x_{34}+x_{24}x_{35},\\ i=x_{35}x_{41}+x_{31}x_{45},j=x_{35}x_{42}+x_{32}x_{45},k=\gamma_{3}x_{45}+x_{35}x_{43},l=\gamma_{4}x_{35}+x_{34}x_{45},\\ m=\gamma_{5}x_{41}+x_{45}x_{51},n=\gamma_{5}x_{42}+x_{42}x_{52},o=\gamma_{5}x_{43}+x_{45}x_{53},p=\gamma_{4}\gamma_{5}+x_{45}x_{54},\\ q=\sqrt{\gamma_{o1}}x_{25}-\sqrt{\gamma_{o2}}x_{15},r=\sqrt{\gamma_{o2}}x_{35}-\sqrt{\gamma_{o3}}x_{25},s=\sqrt{\gamma_{o3}}x_{45}-\sqrt{\gamma_{o4}}x_{35},t=\sqrt{\gamma_{o4}}\gamma_{5}-\sqrt{\gamma_{o5}}x_{45}, \end{array}$$

(25) $$x_{mn}=\sqrt{\gamma_{om}\gamma_{on}}+i\mu_{mn}(m,n=1,2,3,4,5;m\neq n).$$

Theoretical transmission characteristics closely associated with the proposed graphene metamaterial structure can be mathematically expressed as $T=|t{|}^{2}$.

## 5. Analysis and Discussion

**Figure 10:**
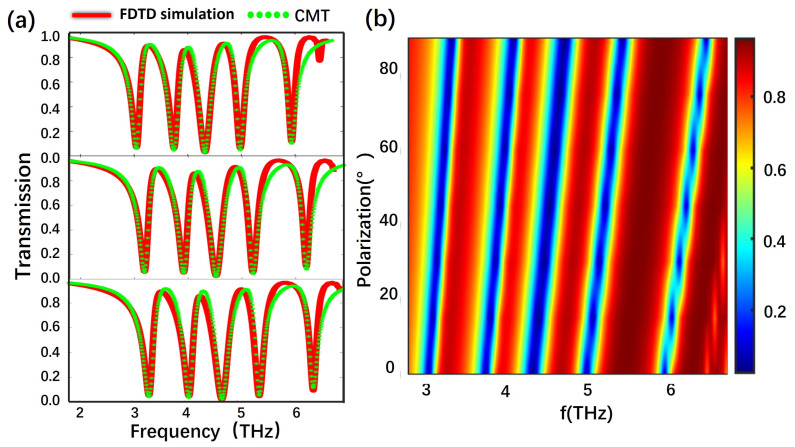

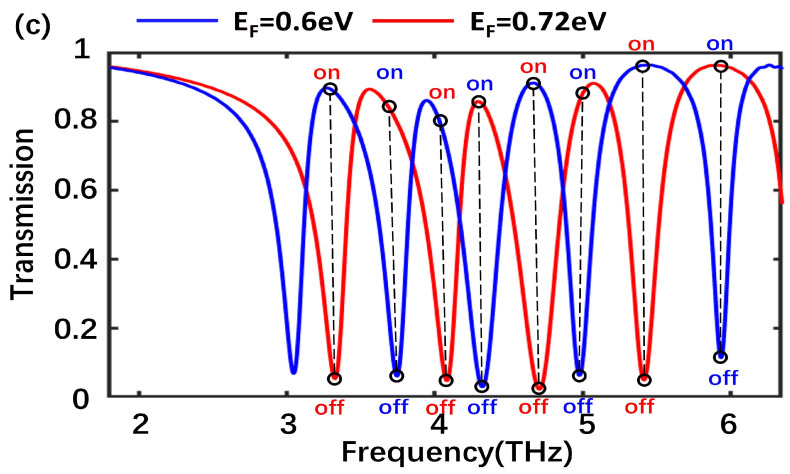
(**a**) The simulated transmission spectrum obtained from FDTD at different E_F_. (**b**) The theoretical transmission spectrum as E_F_ undergoes continuous variation within the range of 0.6 eV to 0.72 eV in FDTD simulation. (**c**) The octa-frequency switch of the transmission spectrum under E_F_ values of 0.60 eV and 0.72 eV, respectively.

As is well known, graphene exhibits satisfactory dynamic tunability. The gate voltage dependency enables dynamic tuning of the transparency window without altering the geometric structure of graphene [45,46,47]. From Figure 10a,b, it becomes evident that the transmission spectra exhibit a nearly linear blue shift with the increase in the Fermi level from 0.6 eV to 0.72 eV. The correlation between the bias voltage and Fermi levels is described by the following equation [30,48]:

(26) $$E_{F}=\hbar v_{F}{\left(\frac{\pi \varepsilon_{0}\varepsilon_{d}V_{g}}{d_{c}e}\right)}^{\frac{1}{2}}{}_{{}^{{}^{}}}$$

where $v_{F}$, $V_{g}$, $\varepsilon_{0}$, $\varepsilon_{d}$, and $d_{c}$ correspond to the Fermi carrier velocity, the gate voltage, the relative dielectric constant of air, the relative dielectric constant of silicon and the thickness of silicon, respectively.

The commonly used metrics for evaluating the performance of optical switch modulators are the MD [49] and the IL [50], which can be obtained as follows:

(27) $$MD=\frac{\left|T_{\max}-T_{\min}\right|}{T_{\max}}\times 100\%$$

(28) $$\mathrm{IL}=-{10\mathrm{lgT}}_{\max}$$

where $T_{\max}$ signifies the maximum transmission and $T_{\min}$ corresponds to the minimum transmission. 

The transmission spectra curve, acquired via FDTD simulations, is depicted in Figure 10a. With an elevation of the graphene Fermi level from 0.6 eV to 0.72 eV, a noticeable blue shift becomes apparent along the red curve. The green dashed line represents the results fitted using the CMT theory, which closely matches the red curve. To observe the blue shift more clearly, the variation process with the increment of the $E_{F}$ is illustrated in Figure 10b. It is shown that the functional relationship between the frequency and transmittance of the structure changes as $E_{F}$ changes continuously within a certain range. The red curve represents the baseline transmission spectra of the quadruple PIT at 0.6 eV. Then, the overall graphene structure’s $E_{F}$ is adjusted to 0.72 eV. As demonstrated in Figure 10c, when the $E_{F}$ is 0.6 eV, the optical switch is considered “off” at the frequency points of 3.74 THz, 4.32 THz, 4.98 THz, and 5.94 THz, while it is “on” at the frequency points of 3.32 THz, 4.08 THz, 4.70 THz, and 5.41 THz. After modulation, the original “off” states at the frequency points of 3.74 THz, 4.32 THz, 4.98 THz, and 5.94 THz change to the “on” states. Conversely, at frequency points of 3.32 THz, 4.08 THz, 4.70 THz, and 5.41 THz, they change to the “off”. Before and after the modulation, the “on” and “off” states are completely reversed. Therefore, the influence of this optical switch is asynchronous.

The MD values and IL values in Figure 10c are 94.0%, 92.48%, 93.54%, 96.54%, 97.51%, 92.86%, 94.82%, and 88.20%, and 0.52 dB, 0.98 dB, 1.37 dB, 0.70 dB, 0.43 dB, 0.63 dB, 0.16 dB, and 0.17 dB, respectively. Here, to demonstrate the superior performance of our proposed modulator, we compare it with several graphene-based metamaterial structures used for optical switch modulators, as shown in Table 1. Clearly, the proposed octa-frequency switch modulator exhibits excellent MD and lower IL performances, providing important guidance for the switch modulation at terahertz frequencies. Overall, this structure exhibits dynamic and controllable spectral response characteristics. Through modulation of the entire structure’s Fermi level, an octa-frequency asynchronous switch can be achieved with an MD of up to 97.51% and excellent modulation performance. 

## 6. Conclusions

In essence, our study introduces a dual-layer graphene terahertz metamaterial structure to realize an octa-frequency asynchronous switch, since quadruple PIT transparency windows have been generated. The mechanism of PIT formation is studied by investigating the electric field distribution. The results derived from the FDTD simulation and CMT fitting show high consistency. Through the manipulation of the Fermi level in graphene, dynamic and controllable multi-switch modulation functionality is achieved. For the proposed octa-frequency switch, the MD is generally greater than 88%, and the IL (0.16 dB < IL < 1.37 dB) is also remarkable. In the case of $E_{F}$ = 0.6 eV ($E_{F}$ = 0.72 eV), the maximum MD reaches 97.51%, corresponding to an IL of 0.40 dB. Moreover, the minimum IL of 0.16 dB corresponds to an MD of 94.82%. Compared to other structures based on PIT, our proposed dynamically controllable optical switch exhibits excellent modulation performance in terms of MD. Therefore, we believe that this structure offers a fresh avenue for exploring optical switches and modulators at terahertz frequency.

## Figures and Tables

**Table 1:** Comparison of graphene-based optical switch performance.

Reference/Year	Material Structure	Modulation Mode	MD (%)	IL (dB and %)	Modulation Band
[49]/2020	Multilayer graphene metamaterials	Multiple frequency	87.8%	0.31 dB	Terahertz
			77.7%	0.20 dB	
[44]/2020	Multilayer graphene metamaterials	Single frequency	83.3%	0.33 dB	Terahertz
[51]/2020	Multilayer graphene metamaterials	Single frequency	83.3%	7.2%	Terahertz
[52]/2020	Single-layer patterned graphene	Dual frequency	93.0%	0.32 dB	Terahertz
			85.0%	0.25 dB	
[53]/2021	Bilayer graphene metamaterials	Triple frequency	86.1%	8.1%	Terahertz
This work	Double-layer patterned graphene	Octa-frequency	94.00%	0.52 dB	Terahertz
			92.48%	0.98 dB	
			93.54%	1.37 dB	
			96.54%	0.70 dB	
			97.51%	0.43 dB	
			92.86%	0.63 dB	
			94.82%	0.16 dB	
			88.20%	0.17 dB	

## Data Availability

The data presented in this study are available on request from the corresponding author. The data are not publicly available due to privacy.
